# Computed exercise plasma lactate concentrations: A conversion formula

**DOI:** 10.1016/j.plabm.2015.11.002

**Published:** 2015-11-28

**Authors:** Lia Bally, Thomas Zueger, Christoph Stettler, Alexander Benedikt Leichtle

**Affiliations:** a Division of Endocrinology, Diabetes & Clinical Nutrition, Inselspital, University Hospital Bern, Switzerland; b Center of Laboratory Medicine, Inselspital, University Hospital Bern, Switzerland

**Keywords:** Lactate, Plasma vs whole-blood, Hematocrit, Enzyme electrodes, Sample treatment

## Abstract

**Objectives:**

Blood lactate measurements are common as a marker of skeletal muscle metabolism in sport medicine. Due to the close equilibrium between the extracellular and intramyocellular space, plasma lactate is a more accurate estimate of muscle lactate. However, whole blood-based lactate measurements are more convenient in field use. The purpose of this investigation was therefore (1) to establish a plasma-converting lactate formula for field use, and (2) to validate the computed plasma lactate levels by comparison to a laboratory standard method.

**Design and methods:**

A total of 91 venous samples were taken from 6 individuals with type 1 diabetes during resting and exercise conditions and assessed for whole blood and plasma lactate using the YSI 2300 analyzer. A linear model was applied to establish a formula for converting whole blood lactate to plasma lactate. The validity of computed plasma lactate values was assessed by comparison to a laboratory standard method.

**Results:**

Whole blood YSI lactate could be converted to plasma YSI values (slope 1.66, intercept 0.12) for samples with normal hematocrit. Computed plasma levels compared to values determined by the laboratory standard method using Passing-Bablok regression yielded a slope of 1.03 (95%CI:0.99:1.08) with an intercept of -0.11 (95%CI:-0.18:-0.06).

**Conclusions:**

Whole blood YSI lactate values can be reliably converted into plasma values which are in line with laboratory determined plasma measurements.

## 1 Introduction

Blood lactate is an important parameter in sports physiology experiments, many of which take place in the field. Due to the lack of laboratory facilities for sample preparation in field settings, assessment of blood lactate concentrations is mostly based on whole blood measurements. However, plasma concentrations are considered to be a better estimate of muscle lactate due to the close equilibrium between the intramyocellular and extracellular compartment [1]. The YSI 2300 analyzer is considered to be a rapid, accurate and reliable measurement device and is therefore commonly used for field testing of lactate in non-hemolyzed whole-blood specimens. The instrument determines extracellular fluid lactate as intracellular lactate is not accessible to the measurement electrode [1], [2], [3]. It is well known that the extracellular fluid concentration displayed on the instrument does not represent the plasma lactate concentration. This discrepancy results from the fact that the YSI 2300 analyzer employs a volume-dependent measurement method, and the erythrocytes exert a diluting effect [4]. As a consequence, the assessed extracellular lactate concentration is always lower than the measured value derived from centrifuged plasma. Computing plasma lactate values by means of a whole blood-based formula would be advantageous in a field study setting often lacking laboratory facilities. However, the manufacturer's literature states there is no reliable way to convert whole blood to plasma lactate values [3].

The purpose of this investigation was therefore to compare whole blood and plasma lactate values obtained with the YSI 2300 analyzer and to establish a conversion formula within a clinically relevant range of lactate concentrations. We also aimed to validate the conversion formula by comparing the computed plasma values with actual plasma measurements using a standard laboratory method.

## 2 Methods

### 2.1 Study procedures

This work was part of a study assessing exercise-related fuel metabolism in individuals with type 1 diabetes, approved by the local ethics committee and registered on www.trialregister.nl (NTR02068638). Blood samples were taken from 6 young male adults with well controlled (mean haemoglobin A1c [±SD] 7.0±0.6%) and complication-free type 1 diabetes both at rest and during exercise conditions designed to elicit lactate concentrations that could be classified as low (0.5–4 mM), moderate (4-8 mM) and high (>8 mM). Exercise modality was 90 min of intermittent intensity cycling (baseline intensity at 50% VO_2_max with interspersed all out 10 s sprints every 10 min). Lithium heparinised blood specimens were collected from an indwelling catheter in the antecubital vein before, during, and after the sprints. Whole blood samples were immediately measured using the YSI 2300 analyzer and then centrifuged. The plasma obtained was kept on ice and analyzed by the YSI 2300 and laboratory reference method within the next 30 minutes.

## 3 Instruments

Whole-blood and plasma lactate was measured using the YSI 2300 glucose and lactate analyzer from Yellow Springs Instruments (Yellow Springs, OH, USA), which utilizes membrane-bound enzyme electrochemical technology. Validation of computed plasma values based on YSI 2300 whole-blood measurements was performed by comparison with the standard laboratory method for plasma lactate on the Roche MODULAR analyzer (Roche Diagnostics AG, Rotkreuz, Switzerland). Both instruments utilize the l-lactate oxidase reaction, which catalyzes the conversion of l-lactate into pyruvate and hydrogen peroxide. Whereas the lactate concentration in the YSI 2300 analyzer is detected by measuring an electric current, the MODULAR instrument detects the resulting color reaction. Both analyzers were calibrated routinely according to the manufacturer's recommendations, and met all quality assurance performance standards during the study period. The MODULAR method was standardized against primary reference material.

## 4 Data analysis

The *conversion* formula for whole blood lactate to plasma lactate was computed by fitting a linear model using a Graphics Processing Unit (GPU) enabled QR decomposition [5] from the YSI whole blood and plasma lactate measurements, followed by the computation of bias-corrected and accelerated (BCa) bootstrap confidence intervals (R=1000). This approach was applied since QR decomposition (decomposition of the data matrix in an orthogonal matrix Q and an upper triangular matrix R) offers in comparison with e.g. Singular Value Decomposition (SVD) or Cholesky decomposition the best balance between efficiency and precision [6], [7]. In addition, inverting an upper triangular matrix is easier and less prone to error than is inverting a sum of squares matrix [7]. *Comparison* between the different methods of lactate determination were performed using Passing-Bablok linear regression [8], [9]. Kendall's rank correlation coefficient was used to determine the degree of association between lactate concentrations derived from the different assessment methods. The level of agreement was assessed using the methods described by Bland and Altman [10]. All statistical analysis were performed using the gputools and Rcmdr (with plugin KMggplot2) packages of the statistical software R 3.1.2 (R Foundation for Statistical Computing, Vienna, Austria).

## 5 Results

A total of 91 samples were included in the analysis. Hematocrit was within the reference range (0.40–0.50 for men) for all except 1 participant who had a hematocrit of 0.55 (hematocrit range=0.42–0.55; median=0.46). Comparison between whole blood and plasma lactate values obtained by the YSI 2300 analyzer showed good correlation (Kendall's tau=0.96) and yielded the following QR decomposition-based conversion equation:

plasmalactate=1.66×wholebloodlactate+0.12

with a 95% confidence interval (CI) for the intercept of 0.0741–0.1896 and for the slope of 1.633–1.707.

**Fig. 1:**
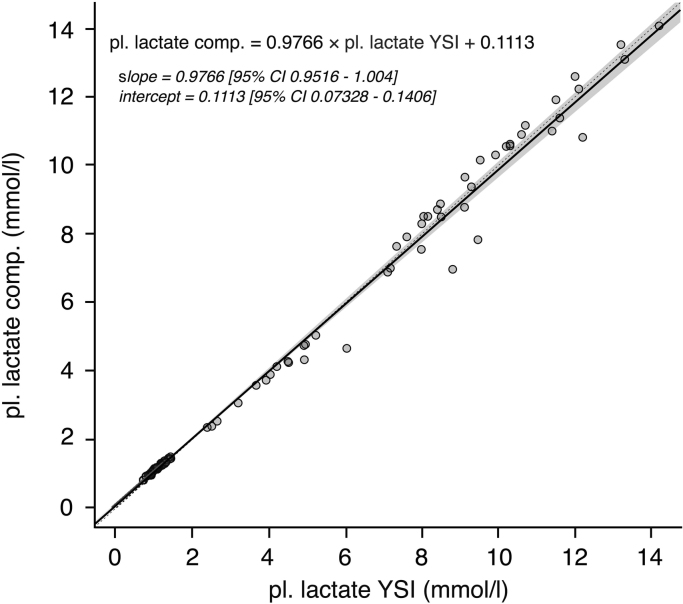
Passing-Bablok regression analysis for the comparison between measured YSI plasma lactate concentration (pl. lactate YSI) and computed plasma lactate concentration (pl. lactate comp.) based on YSI whole blood measurements. The solid black line represents the regression line, the dashed line indicates the identity line and the grey zone represents the 95% confidence interval (CI) of the regression line. Regression equation and 95% CIs for slope and y-intercept are presented. Figure 1 in Bally et al. [11]*. Bland-Altman for plasma lactate concentrations obtained from YSI (pl. lactate YSI) versus computed plasma lactate values from YSI whole blood measurements (pl. lactate comp). Dotted lines represent limits of agreement (±1.96 times the standard deviation of the bias)*.

**Fig. 2:**
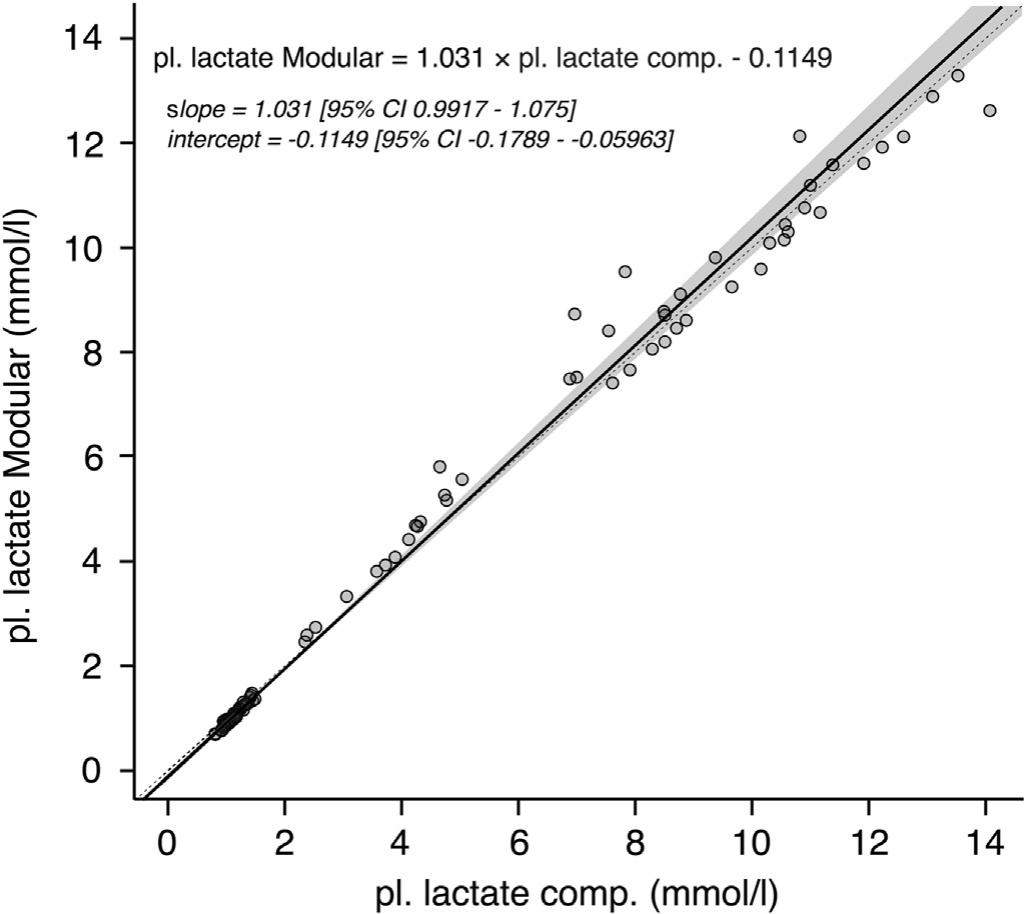
Passing-Bablok regression analysis for the comparison between computed YSI plasma lactate values (pl. lactate computed) and plasma lactate concentrations determined by the laboratory reference instrument MODULAR (pl. lactate Modular). Linear regression is represented by the solid black line, the dashed line indicates the line of identity and the grey zone represents the 95%CI. Regression equation and 95% CIs for slope and y-intercept are presented. Figure 2 in Bally et al. [11]. *Bland-Altman plot for computed YSI plasma concentrations (pl. lactate comp) versus measured plasma concentration using the MODULAR laboratory instrument (pl. lactate Modular). Dotted lines represent limits of agreement (±1.96 times the standard deviation around the bias)*.

Computed plasma lactate values based on the conversion formula correlated well with YSI 2300 measured plasma lactate concentrations (tau=0.96) and regression analysis (see Fig. 1) revealed a slope of 0.98 (95%CI=0.95:1.00) and an intercept of 0.11 (95%CI=0.07:0.14). Bland-Altman analysis showed limits of agreement between −0.80 mmol/l and 0.80 mmol/l (see Fig. 1 in Data in Brief article: Bally et al. [11]). The correlation between the computed YSI plasma lactate values and the lactate measurements obtained from the laboratory method (MODULAR) is shown in Fig. 2 (slope=1.03, 95%CI=0.99:1.08; intercept=−0.11, 95%CI=−0.18−:−0.06, tau=0.94)). The levels of agreement between the computation method and the laboratory method are shown in Fig. 2 in the Data in Brief article [11]. The mean bias was −0.04 mmol/l and the 95% limits of agreement were −0.91 to 0.83 mmol/l.

## 6 Discussion

This study investigated whether YSI-based whole blood lactate values can be reliably converted into plasma lactate values in order to avoid time- and cost-intensive sample preparation.

The results of our Passing-Bablok regression analysis between computed and reference lactate values indicate that the fixed bias is clinically negligible (<0.2 mmol/L) and no proportional bias exists [12]. This is supported by the high level of agreement as shown using Bland-Altman analysis.

The suggested conversion formula therefore enables valid plasma lactate estimates across a clinically relevant range of blood lactate concentrations in the population studied, avoiding the need for cumbersome sample preparation and laboratory facilities. In contrast to the formula presented by Wandrup et al. [13], our formula does not include the hematocrit. This might be advantageous for e.g. sports medicine and field testing, as (presumably) healthy athletes under supervised training should have hematocrit values within the reference range (for men 0.40–0.50). In these very common applications of POC field testing, the ability to calculate plasma lactate without needing to determine additional the hematocrit saves time and effort. However, under very extreme conditions (e.g. heat, inadequate fluid intake) and with subjects who are not in good health, the formula might not apply. In these cases, determination of the hematocrit should be considered to achieve proper conversion using the Wandrup formula.

Despite the well-known differences between plasma and whole blood lactate values obtained with YSI 2300 analyzer [14], [15], the validity of a plasma lactate conversion formula which does not require hematocrit determination has not hitherto been investigated by comparison to a standard laboratory method.

In contrast to the manufacturer's literature, which precludes a reliable conversion of whole blood to plasma lactate [3], our findings suggest excellent agreement between plasma lactate values derived from the conversion formula and measured with a standard laboratory method. Since the MODULAR method used as the reference procedure is highly precise (intra- and inter-day CV<1.5% [16] over the whole clinically relevant range) and standardized against primary standard material, although the measurement uncertainty of the computed plasma lactate equivalent is not directly traceable, it is also presumably low in view of the good agreement between the methods. Several previous studies reported comparable levels of agreement when assessing point-of-care whole blood lactate analyzers against laboratory reference methods [17], [18], [19], however none of them investigated computed plasma lactate levels.

As all our subjects showed hematocrit values within or near to the reference range (±10%), it is likely that our conversion formula cannot be used for patients with anemia or polycythemia due to the differing contribution of red blood cells to overall specimen volume. Additionally, hemolysis and preanalytical problems (squeezing, contamination with disinfectant, etc.) have to be considered. For example, we could trace five outlier samples (numbers 61–65 in the Data in Brief article [11]) to one sampling, which may have been subject to greater preanalytical variation. Since the influence of these samples on the final conversion formula was negligible and they were representative of unavoidable variability in a difficult field sampling setting, we decided not to remove them from the dataset. However, as conversion and comparison were based on the same sampling set, both can only show variation that was present in the sample set. If applied to independent samples, comparisons might yield altered bias estimates. We collected blood from well controlled physically active patients with type 1 diabetes for our investigation. It is unlikely that this accounts for any differences compared to a non-diabetic population, since there is no evidence that any lactate measurement method is influenced by diabetes. Furthermore blood glucose levels were in a nearly euglycemic range (5-9 mmol/L) during lactate assessment.

In summary, computed plasma lactate values based on whole blood YSI lactate measurements in individuals with normal or near normal hematocrits showed excellent agreement with a standard laboratory method. The proposed conversion formula enables plasma lactate determination without the need for time- and cost-intensive sample preparation, which is particularly advantageous for exercise studies in the field requiring high flexibility.

## Declaration of interest

The authors declare that there is no conflict of interest.

## Funding/Grants

This study was supported by unrestricted Grants from the Swiss National Science Foundation (Grant number 320030_149321/1)
