# Supplementary material for: Computed exercise plasma lactate concentrations: A conversion formula
Source: Pract Lab Med. 2015 Nov 28;4:11–5. doi: 10.1016/j.plabm.2015.11.002 (PMC5574503; doi:10.1016/j.plabm.2015.11.002)
Supplement: Supplementary file 1 — Supplementary material [file mmc1.zip › Data in Brief/Data in Brief.docx]

*Data article*

**Title: *Method comparison and computational conversion: YSI whole-blood lactate, plasma lactate computed from whole blood concentrations, and laboratory plasma lactate reference measurements.***

**Authors: Lia Bally (1), Thomas Zueger (1), Christoph Stettler (1), Alexander Benedikt Leichtle (2)**

**Affiliations:** **Division of Endocrinology, Diabetes & Clinical Nutrition (1) and University Institute of Clinical Chemistry (2), Inselspital- Bern University Hospital, Bern, Switzerland**

**Contact email: alexander.leichtle@insel.ch**

**Abstract**

The present data file contains lactate measurements (in mmol/L) from individuals with type 1 diabetes under physical exercise. Lithium-heparin whole blood samples were collected and measured with the YSI 2300 Stat plus Lactate analyzer directly, after centrifugation, and with a laboratory reference method for plasma. In a corresponding research article [1], we established a conversion fromula from YSI whole blood lactate to computed plasma lactate and compared the converted values to a laboratory reference method (Roche MODULAR). Bland-Altman plots for YSI plasma lactate vs. computed plasma lactate and computed plasma lactate vs. MODULAR plasma lactate are included in this *Data in Brief*.

**Specifications Table** *[please fill in right-hand column of the table below]*

| Subject area | *Medicine* |
| --- | --- |
| More specific subject area | *Sports and Laboratory Medicine* |
| Type of data | *Table, Bland-Altman plots* |
| How data was acquired | *Whole blood lactate measurements (YSI 2300 Stat plus analyzer) and plasma lactate measurements (YSI YSI 2300 Stat plus analyzer and Roche MODULAR platform)* |
| Data format | *Comma separated value table (.csv), JPEG figures* |
| Experimental factors | *Brief description of any pretreatment of samples* |
| Experimental features | *Whole blood samples from type 1 diabetics under exercise were acquired and lactate was determined in whole blood and plasma* |
| Data source location | *Inselspital – Bern University Hospital, Bern, Switzerland* |
| Data accessibility | *Attached CSV file and JPEG figures* |

**Value of the data**

This dataset contains data from a method comparison study including two different preanalytical preparation methods (whole blood and plasma) and two different analytical platforms (YSI and Roche MODULAR), all from the same set of 91 exercise samples, that account for values over the whole clinically relevant range. It also includes computed values generated by QR decomposition-based regression.

This dataset might exemplarily be used for

- studies jointly assessing different sample materials and methods.
- studies comparing different regression approaches
- studies addressing three-class separation problems
- teaching of method comparison and conversion.

**Data**

The data table (CSV format with point as decimal and semicolon as column separator, “Inselpital_Plasma_Lactate.csv”) in this *Data in Brief* contains five columns:

sample: the anonymized sample identifier

wb_lact_YSI: Lactate measurements from whole blood with the YSI 2300 Stat plus analyzer (mmol/L)

plasma_lact_YSI: Lactate measurements from plasma with the YSI 2300 Stat plus analyzer (mmol/L)

plasma_lact_MODULAR: Lactate measurements from plasma with the Roche MODULAR platform (mmol/L)

plasma_lact_YSI_comp: Plasma lactate levels computed from “wb_lact_YSI” using the conversion formula from Bally et al. [1] (corresponds to mmol/L).

Additionally, two figures are included:

Figure 1: Bland-Altman plots for YSI plasma lactate vs. computed plasma lactate

Figure legend: *Bland-Altman for plasma lactate concentrations obtained from YSI (pl. lactate YSI) versus computed plasma lactate values from YSI whole-blood measurements (pl. lactate comp). Dotted lines represent limits of agreement (± 1.96 times the standard deviation of the bias).*

Figure 2: Bland-Altman plots for computed plasma lactate vs. MODULAR plasma lactate

Figure legend: *Bland-Altman plot for computed YSI plasma concentrations (pl. lactate comp) versus measured plasma concentration using the Modular reference instrument (pl. lactate Modular). Dotted lines represent limits of agreement (± 1.96 times the standard deviation around the bias).*

Samples № 61 to 65 arise all from the same potentially preanalytically biased sampling and might be considered as “outliers”.

**Experimental Design, Materials and Methods**

This anonymized data set arises from a trial assessing exercise-related fuel metabolism in individuals with type 1 diabetes. The trial was approved by the local ethics committee and registered on [www.trialregister.nl](http://www.trialregister.nl) (NTR02068638). 91 blood samples were taken from 6 young male adults with type 1 diabetes during rest and exercise to elicit lactate concentrations varying in a clinically relevant range. Lithium heparin whole blood was collected and measured with the YSI 2300 Stat plus lactate analyzer directly, after centrifugation, and with a the Roche MODULAR platform as a laboratory reference method for plasma lactate determination. We established a conversion formula from YSI whole blood lactate to computed plasma lactate by QR decomposition-based regression [2-4]:

*𝑝𝑙𝑎𝑠𝑚𝑎 𝑙𝑎𝑐𝑡𝑎𝑡𝑒=1.66×𝑤ℎ𝑜𝑙𝑒 𝑏𝑙𝑜𝑜𝑑 𝑙𝑎𝑐𝑡𝑎𝑡𝑒+0.12, with a 95% CI for the intercept of 0.0741 – 0.1896 and for the slope of 1.633 – 1.707.*

We compared the converted values to a laboratory reference method (Roche MODULAR). Bland-Altman plots for YSI plasma lactate vs. computed plasma lactate and computed plasma lactate vs. MODULAR plasma lactate are included in this *Data in Brief*

**Acknowledgements** The authors would like to thank the volunteers for their participation.

**Funding:** This study was supported by unrestricted grants from the Swiss National Science Foundation (grant number 320030_149321/1)

**References**

[1] L. Bally, T. Zueger, C. Stettler, A.B. Leichtle, Computed exercise plasma lactate concentrations: a conversion formula, Practical Laboratory Medicine. (in press).

[2] J. Buckner, M. Seligman, J. Wilson, RPackage “gputools,” (2014) 1–29.

[3] G.A.F. Seber, A.J. Lee, Linear Regression Analysis, John Wiley & Sons, Hoboken, NJ, USA, 2012. doi:10.1002/9780471722199.

[4] J.D. Brown, Linear Models in Matrix Form, Springer, Cham, 2015. doi:10.1007/978-3-319-11734-8.
