# Supplementary figures and images for: Computed exercise plasma lactate concentrations: A conversion formula
Source: Pract Lab Med. 2015 Nov 28;4:11–5. doi: 10.1016/j.plabm.2015.11.002 (PMC5574503; doi:10.1016/j.plabm.2015.11.002)

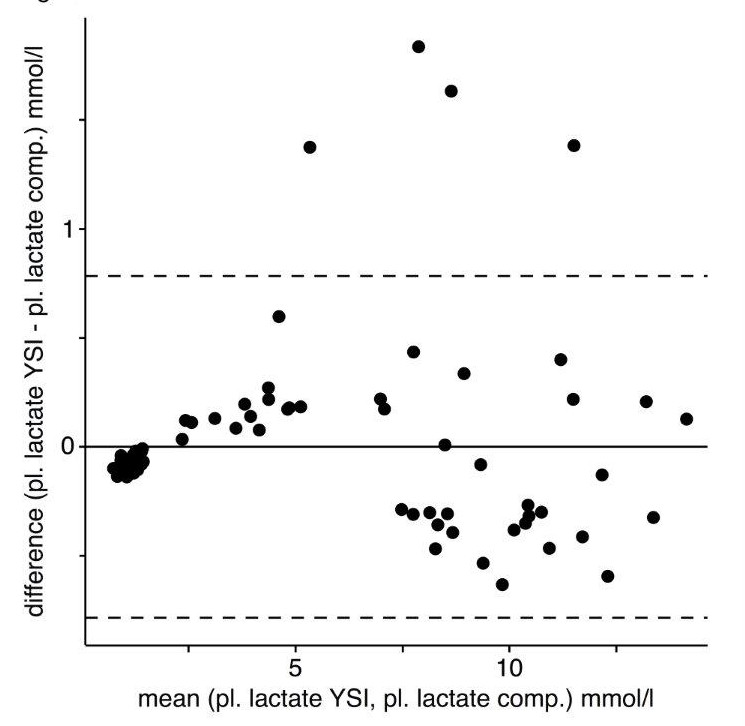

Supplement: Supplementary file 1 — Supplementary material [file mmc1.zip › Data in Brief/Figure 1.jpg]

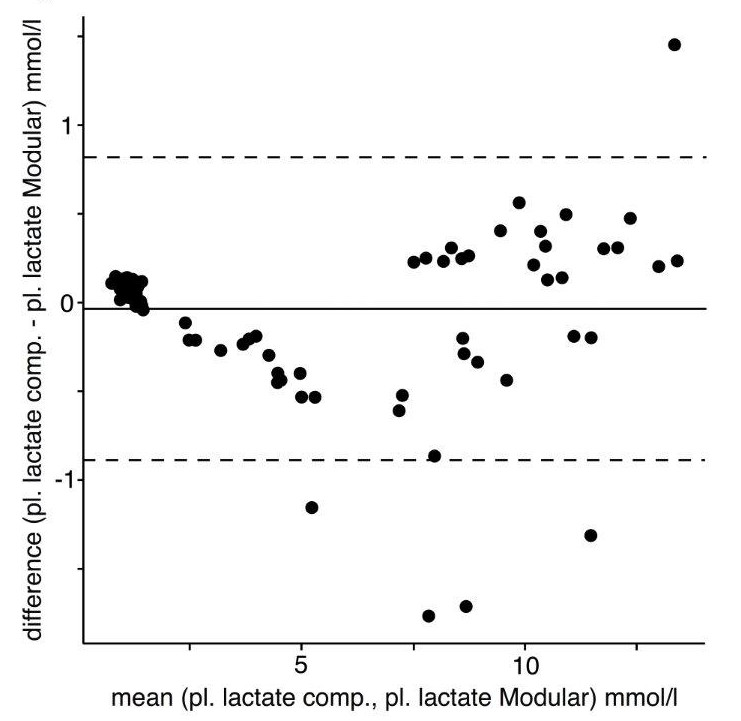

Supplement: Supplementary file 1 — Supplementary material [file mmc1.zip › Data in Brief/Figure 2.jpg]
